# When substrate inhibits and inhibitor activates: implications of β-glucosidases

**DOI:** 10.1186/s13068-016-0690-z

**Published:** 2017-01-03

**Authors:** Silja Kuusk, Priit Väljamäe

**Affiliations:** Institute of Molecular and Cell Biology, University of Tartu, Riia 23b – 202, 51010 Tartu, Estonia

**Keywords:** β-glucosidase, Glucose tolerance, Glucose inhibition, Glucose activation, Nonproductive binding, Transglycosylation

## Abstract

**Background:**

β-glucosidases (BGs) catalyze the hydrolysis of β-glycosidic bonds in glucose derivatives. They constitute an important group of enzymes with biotechnological interest like supporting cellulases in degradation of lignocellulose to fermentable sugars. In the latter context, the glucose tolerant BGs are of particular interest. These BGs often show peculiar kinetics, including inhibitory effects of substrates and activating effects of inhibitors, such as glucose or xylose. The mechanisms behind the activating/inhibiting effects are poorly understood. The nonproductive binding of substrate is expected in cases where enzymes with multiple consecutive binding subsites are studied on substrates with a low degree of polymerization. The effects of inhibitors to BGs exerting nonproductive binding of substrate have not been discussed in the literature before.

**Results:**

Here, we performed analyses of different reaction schemes using the catalysis by retaining BGs as a model. We found that simple competition of inhibitor with nonproductive binding of substrate can account for the activation of enzyme by inhibitor without involving any allosteric effects. The transglycosylation to inhibitor was also able to explain the activating effect of inhibitor. For both mechanisms, the activation was caused by the increase of *k*_cat_ with increasing inhibitor concentration, while *k*_cat_/*K*_m_ always decreased. Therefore, the activation by inhibitor was more pronounced at high substrate concentrations. The possible contribution of the two mechanisms in the activation by inhibitor was dependent on the rate-limiting step of glycosidic bond hydrolysis as well as on whether and which glucose-unit-binding subsites are interacting.

**Conclusion:**

Knowledge on the mechanisms of the activating/inhibiting effects of inhibitors helps the rational engineering and selection of BGs for biotechnological applications. Provided that the catalysis is consistent with the reaction schemes addressed here and underlying assumptions, the mechanism of activation by inhibitor reported here is applicable for all enzymes exerting nonproductive binding of substrate.

**Electronic supplementary material:**

The online version of this article (doi:10.1186/s13068-016-0690-z) contains supplementary material, which is available to authorized users.

## Background

β-glucosidases (BGs) catalyze the hydrolysis of β-glycosidic bond in glucosyl derivatives like aryl-glucosides, cellobiose, or higher cellooligosaccharides. BGs are an important group of enzymes with biological as well as biotechnological interest [1]. In the road towards green and sustainable energy, the enzymatic hydrolysis of lignocellulose rich biomass to fermentable sugars has been in focus of intensive research during the last decade. For economic reasons, the enzymatic hydrolysis of lignocellulose is conducted at high dry matter consistency. An inevitable consequence of this is the accumulation of the cellulose hydrolysis products at high concentrations, which, in turn, results in the feedback inhibition of cellulases [2, 3]. The most sensitive to product inhibition are processive cellobiohydrolases, which are inhibited by their own product—cellobiose. On the other hand, glucose is a rather weak inhibitor of cellobiohydrolases [4, 5]. By degrading cellobiose into two molecules of glucose, BGs relieve the inhibition of cellobiohydrolases and accelerate the overall rate of lignocellulose conversion. However, the product inhibition of BGs by glucose eventually results in the accumulation of cellobiose and inhibition of cellobiohydrolases. Therefore, a suitable BG candidate for supporting cellulases in lignocellulose conversion should, besides having high catalytic efficiency, be tolerant to glucose inhibition [6]. Due to this, a lot of efforts have been made in searching and engineering glucose tolerant BGs. Glucose tolerant BGs usually have half-inhibiting glucose concentrations in a molar or sub-molar range. They mostly belong to the family 1 of glycoside hydrolases (GHs) [7]. Kinetic studies of BGs are often complicated by non Michaelis–Menten kinetics, which reveals as a decrease in the rate of substrate degradation at substrate concentrations higher than optimum. It has been shown that transglycosylation to substrate is responsible for the phenomenon of substrate inhibition [8–10]. Another kinetic peculiarity often observed with glucose tolerant BGs is the activation of enzyme at lower but inhibition at higher inhibitor concentrations. This phenomenon has been often reported with the inhibition of BGs by glucose [11–36], and xylose [13, 15, 19, 21, 22, 28–31, 37], but also by other sugars [19, 28, 31]. With some of these BGs, the Michaelis–Menten saturation kinetics holds [19, 21, 26, 31], with others, it does not [32, 38]. Most common mechanistic interpretations of the activation by inhibitor include transglycosylation to inhibitor [19, 26] and binding of inhibitor to an allosteric regulatory binding site [21, 39], whereas the glucose tolerance has been explained by the deep and narrow active site architecture of GH1 BGs [40]. Since the transglycosylation products of BGs may intervene with the reactions of cellulases as well as other enzymes present in lignocellulolytic enzyme cocktails, a detailed knowledge on the mechanisms behind glucose tolerance and activation is a pre-requisite for the selecting and engineering of BGs.

Nonproductive binding occurs when substrate combines with free enzyme in a way that excludes further productive binding of substrate. Thus, nonproductive binding can be regarded as a competitive inhibition analogue of substrate inhibition. Kinetic consequences of nonproductive binding are the reduced *k*_cat_ and *K*_m_ values with *k*_cat_/*K*_m_ being unaffected [41, 42]. Nonproductive binding is expected in the cases where naturally polymer active enzymes are studied on model substrates with a low degree of polymerization [41]. A structural characteristic of polymer active enzymes is the presence of multiple consecutive monomer-unit binding sites in the active site [43]. If studied with, e.g., a dimeric substrate, the binding of the substrate without the correct positioning for the catalysis seems plausible. A good example of nonproductive binding is provided by the hydrolysis of fluorogenic model substrates by *T. reesei* Cel7A, a processive cellobiohydrolase with the active site tunnel containing 10 glucose-unit-binding sites (−7 to +3) [44, 45]. Here and below, we use the subsite nomenclature recommended by Davies et al. [46]. The enzyme has a 13-fold lower *K*_m_ value for methylumbelliferyl-cellobioside compared to that of methylumbelliferyl-lactoside [47]. However, the *k*_cat_/*K*_m_ values are about equal on both substrates [47]. Low *K*_m_ for methylumbelliferyl-cellobioside is apparently caused by the nonproductive binding to subsites +1/+3 that competes with the productive binding to −2/+1. The strong nonproductive binding of methylumbelliferyl-cellobioside is caused by the strong binding of the cellobiose moiety to the product-binding sites +1/+2 necessary for enzymes processivity [48]. On the other hand, lactose binds to Cel7A with 16.8-fold lower affinity than cellobiose [47] and the nonproductive binding of methylumbelliferyl-lactoside is expected to be much less dominant, if present at all.

Through the analysis of different reaction schemes, using the mechanism of retaining BGs as the model system, we here provide evidence that simple competition of inhibitor with the nonproductive binding of substrate can account for the activating effects of inhibitor. The effects of transglycosylation to inhibitor and to substrate are also discussed.

## Results

### Nonproductive binding of substrate

**Fig. 1:**
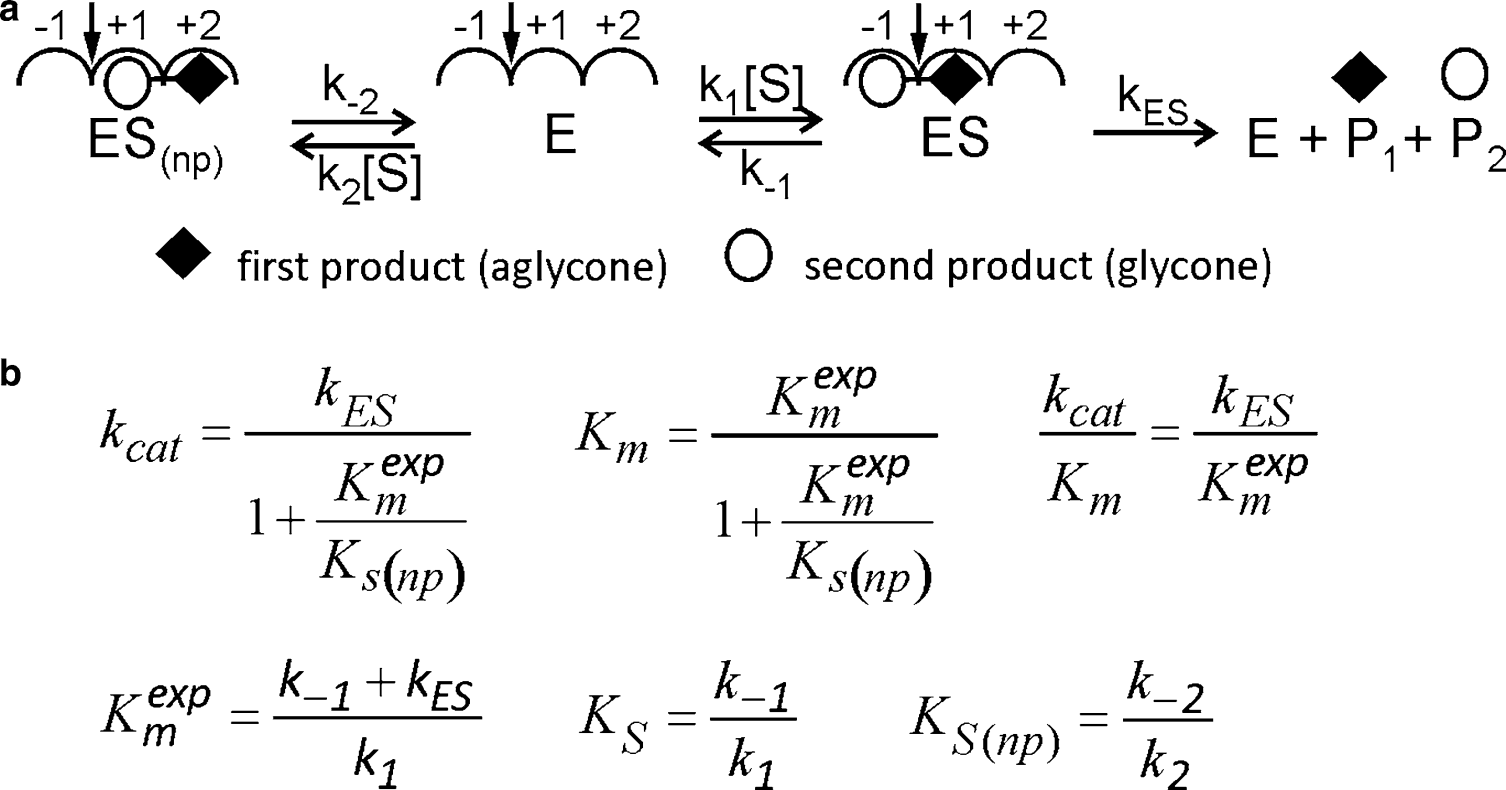
Nonproductive binding of substrate. **a** When a BG containing three consecutive binding subsites, one glycone-binding subsite (−1) and two aglycone-binding subsites (+1 and +2) are studied with a dimeric substrate (like pNPG), the substrate can combine with the enzyme both productively (ES complex) and nonproductively (ES_(np)_ complex). The position of the scissile bond is shown with the* arrow*. **b** Michaelis–Menten equation parameters derived for the mechanism in panel a using the steady-state treatment. *k*_ES_ is the lumped catalytic rate constant for the formation of both products. The superscript ^exp^ refers to the expected value *K*_m_ would have if no nonproductive complexes were formed. *K*_s_ and *K*_s(np)_ are the true equilibrium dissociation constants of productive and nonproductive enzyme-substrate complexes, respectively

Although relevant for a wide group of enzymes, let us inspect the nonproductive binding using BG as an example. At least two binding subsites are required for the catalysis of the hydrolysis of glycosidic bond in glucosyl derivatives. Glucose is bound to subsite −1 and the aglycone leaving group is bound to +1. In the case of oligosaccharide substrates, the aglycone is also a saccharide, but for simplicity, we designate this moiety as the aglycone, regardless (Fig. 1a). This situation is relevant for the hydrolysis of fluoro- or chromogenic model substrates like para-nitrophenol-β-glucoside (pNPG), often used in the studies of BGs. In the case of pNPG substrate, the first product is the aglycone leaving group (para-nitrophenol) and the second is glucose (Fig. 1a). Although the presence of just two subsites is enough for the hydrolysis of aryl-glucosides and cellobiose, many BGs have longer substrate-binding sites with three or more glucose-unit binding subsites [49]. One possible reason for this is the necessity to act on longer oligosaccharide substrates. For the phenomenon of nonproductive binding of a dimeric substrate to occur at least three binding subsites, −1 to +2 must be present. According to the definition, the scissile bond is in between subsites −1 and +1 [46]. The binding of substrate to −1/+1 results in a productive complex, whereas the binding to subsites +1/+2 is nonproductive (Fig. 1a). Applying the steady-state assumption to [ES] results in Eq. 1 that can also be found in textbooks [41, 42]:

1 $$v = \frac{{\left[ S \right]\left[ {E_{0} } \right]\left( {\frac{{k_{\text{ES}} }}{{1 + \frac{{K_{\text{m}}^{\exp } }}{{K_{{{\text{s}}\left( {\text{np}} \right)}} }}}}} \right)}}{{\left[ S \right] + \left( {\frac{{K_{\text{m}}^{\exp } }}{{1 + \frac{{K_{\text{m}}^{\exp } }}{{K_{{{\text{s}}\left( {\text{np}} \right)}} }}}}} \right)}}.$$

*k*_ES_ is the true catalytic constant representing the reaction of the productive enzyme-substrate complex, and *K*_m_^exp^ and *K*_s(np)_ stand for the Michaelis constant (superscript ^exp^ refers to the expected value, the *K*_m_ would have if no nonproductive complexes were formed, i.e., (* k*_−1_ + *k*_ES_)/*k*_1_ for scheme in Fig. 1a [41]) and the equilibrium dissociation constant of the nonproductive enzyme-substrate complex, respectively. Note that when catalysis is slow compared to dissociation (*k*_ES_ ≪ *k*_−1_) *K*_m_^exp^ approaches the value of the true equilibrium dissociation constant of the productive enzyme-substrate complex (*K*_s_), whereas *K*_s(np)_ is always the true equilibrium constant. An important consequence from the Eq. 1 is that Michaelis–Menten kinetics holds also in the presence of the nonproductive binding. This is apparently the reason why the contribution of the nonproductive binding is often overlooked in kinetic studies. Since the same substrate is responsible for the productive and nonproductive binding, the presence of the nonproductive binding is not apparent in simple studies of substrate–velocity relationships. The *k*_cat_ and *K*_m_ values are both reduced by the nonproductive binding. However, since both *k*_cat_ and *K*_m_ are reduced by the same factor, the efficiency constant (*k*_cat_/*K*_m_) is not affected by the nonproductive binding (Fig. 1b).

### Effects of inhibitors to enzymes that have nonproductive binding of substrate

**Fig. 2:**
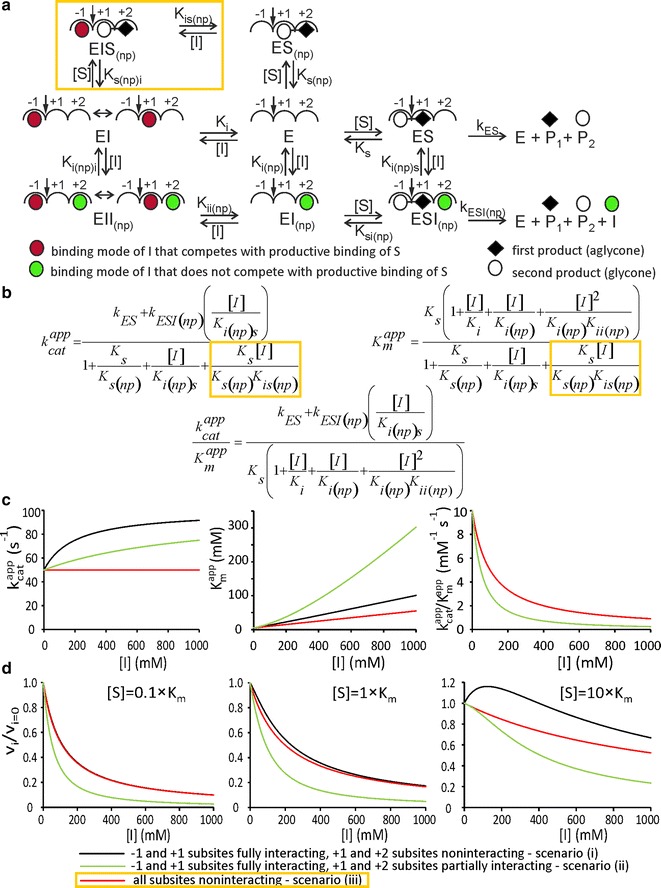
Effect of inhibitor to an enzyme exerting the nonproductive binding of substrate. **a** Binding of inhibitor to binding site +2 [designated as the nonproductive (np) binding mode of inhibitor] competes with the nonproductive binding but not with the productive binding of substrate. Binding of inhibitor to subsites −1 or +1 competes with the productive binding of substrate. For simplicity, these binding modes are lumped together (designated by double-headed* arrow*) in EI and EII_(np)_ complexes. Binding of inhibitor to +1 competes also with the nonproductive binding of substrate, whereas binding to −1 does not. The latter possibility is accounted for by the presence of EIS_(np)_ complex (within *yellow box*). The stability of all complexes is represented by equilibrium dissociation constants. **b** Michaelis–Menten equation (Eq. 2) parameters derived using rapid equilibrium treatment. The same equations are applicable for the formation of the first and the second product as well as for the disappearance of substrate. The terms within *yellow boxes* appear only when EIS_(np)_ complex is included. **c** Dependency of parameters on inhibitor concentration. **d** Ratios of rates measured in the presence (*v*_*i*_) and absence (*v*_*i*=0_) of inhibitor as a function of inhibitor concentration. The concentration of substrate (as a multiple of its *K*_m_ value at [I] = 0) is shown in the plots. The results corresponding to the three scenarios are depicted in panels, c and d. Following values for parameters were used in analyses of all scenarios: *k*_ES_ = *k*_ESI(np)_ = 100 s^−1^, *K*_s_ = *K*_s(np)_ = 10 mM, *K*_i_ = *K*_i(np)_ = *K*_ii(np)_ = *K*_i(np)i_ = 100 mM. In the case of both, first and second scenario, the values of *K*_is(np)_ = *K*_s(np)i_ were set 10^6^ mM. In the first scenario, we used *K*_si(np)_ = 10 mM and *K*_i(np)s_ = 100 mM, whereas in the second scenario, the corresponding values were set to 50 and 500 mM, respectively. The parameter values in the third scenario were as in the first scenario except that *K*_s(np)i_ was set to 10 mM and *K*_is(np)_ to 100 mM

By including a monosaccharide inhibitor (I) to the mechanism in Fig. 1a, we have mechanism depicted in Fig. 2a. Like substrate, inhibitor is allowed to bind to the different binding sites on the enzyme. The binding of the inhibitor to subsite −1 or +1 competes with the productive binding, whereas the binding to subsite +1 or +2 competes with the nonproductive binding of substrate. It is important to note that the binding of the inhibitor to subsite +2 competes with the nonproductive binding but not with the productive binding of substrate (Fig. 2a). Here, we refer to the binding of the inhibitor to subsite +2 as the nonproductive binding mode of inhibitor (EI_(np)_). By allowing ESI_(np)_ complex in Fig. 2a, we assume that subsites +1 and +2 are not fully interacting. In the case of fully interacting subsites, the binding of two ligands to the adjacent subsites is not possible without the covalent bond between the ligands. For example, BGs may accommodate a cellobiose with the glycosidic bond oxygen between subsites +1 and +2, but they may not accommodate two glucose molecules bound to +1 and +2 because of the steric clash between the anomeric beta-OH and the C4-OH of the adjacent glucoses. To stress this possibility, the binding subsites of BGs are often referred to as anhydroglucose-unit binding sites. The plausibility of assuming noninteracting subsites, thus, depends on the spatial flexibility of the subsites of particular enzyme. Often used for BGs [50–56], the subsite mapping method of Hiromi [57–59] assumes noninteracting subsites. The noninteracting subsites model holds in subsite mapping, since it relies on the analysis of *k*_cat_/*K*_m_, a parameter that is independent of the nonproductive binding (see Eq. 1). However, the analysis of the nonproductive binding and inhibition made here depends on whether the subsites are interacting or not.

Solving the mechanism in Fig. 2a using rapid equilibrium treatment results in the rate equation in the form of Michaelis–Menten equation (Eq. 2). We turn to the plausibility and consequences of using rapid equilibrium treatment for the retaining BGs later.

2 $$v = \frac{{\left[ S \right]\left[ {E_{0} } \right]k_{\text{cat}}^{\text{app}} }}{{\left[ S \right] + K_{\text{m}}^{\text{app}} }}.$$

The apparent catalytic constant (*k*_cat_^app^) and the Michaelis constant (*K*_m_^app^) depend on the nonproductive binding and the concentration of inhibitor ([I]) as depicted in Fig. 2b. Note that because of the closed cycles in the reaction scheme, not all the equilibrium dissociation constants defined in Fig. 2a appear in the equations in Fig. 2b. For example, *K*_si(np)_ does not appear in the equations because of the relationship *K*_s_ *K*_i(np)s_ = *K*_si(np)_ *K*_i(np)_, i.e., there are four equilibrium constants, but only three are required to define the closed cycle with four complexes. Since the same products are formed from both productive complexes ES and ESI_(np)_, the apparent parameters of Michaelis–Menten equation are independent of whether the formation of the first (aglycone) or the second product (glycone) or the disappearance of substrate is measured. In Fig. 2a, two possible scenarios are depicted depending on whether the binding subsites adjacent to the scissile bond (−1 and +1) are interacting or not. If subsites −1 and +1 are fully interacting, the formation of EIS_(np)_ complex (framed by the yellow box in Fig. 2a) is not possible and the resulting rate equations are somewhat simpler (Fig. 2b). Figure 2c depicts the dependency of apparent parameters of the Michaelis–Menten equation on inhibitor concentration at three different scenarios. (1) Fully interacting subsites −1 and +1, and noninteracting subsites +1 and +2 [i.e., *K*_s_ = *K*_si(np)_ and *K*_i(np)_ = *K*_i(np)s_]. (2) Fully interacting subsites −1 and +1, and partially interacting subsites +1 and +2 [i.e., *K*_s_ < *K*_si(np)_ and correspondingly *K*_i(np)_ < *K*_i(np)s_]. (3) All subsites are noninteracting (Fig. 2a, complex within yellow box included). In all the cases, the strength of productive and nonproductive binding modes of substrate was taken equal (*K*_s_ = *K*_s(np)_). The same was assumed for the binding modes of inhibitor competing with the productive and nonproductive binding of the substrate (*K*_i_ = *K*_i(np)_). In addition, the rate constants for the product formation were considered independent of the bound inhibitor (*k*_ES_ = *k*_ESI(np)_). Most importantly, the first two scenarios were able to capture the phenomenon of inhibitor being an activator at low and inhibitor at high concentrations. The activating effect of inhibitor was manifested by the increase in the apparent *k*_cat_ with the increasing inhibitor concentration. However, the increase in *K*_m_^app^ counterbalanced the increase in *k*_cat_^app^, so that (*k*_cat_/*K*_m_)^app^ decreased with the increasing inhibitor concentration (Fig. 2c). The activating effect of inhibitor increased with increasing the strength of the nonproductive binding of substrate relative to that of the productive-binding mode. The same was true when the binding of the inhibitor to subsite +2 was stronger than to subsite −1 and/or +1 (Additional file 1: Fig. S1). In case, all the subsites were assumed to be noninteracting the activating effect of inhibitor revealed only when the nonproductive-binding modes were stronger than the productive binding modes (*K*_s_ > *K*_s(np)_ and/or *K*_i_ > *K*_i(np)_). This situation seems unlikely with native substrates but may occur with artificial model substrates. In case, all the subsites are fully interacting, the activation by inhibitor is not possible for the mechanism depicted in Fig. 2a. This is because all the binding modes of inhibitor that compete with the nonproductive binding also compete with the productive binding (ESI_(np)_ is not possible). However, the ESI_(np)_ complex can be introduced while keeping the assumption of the fully interacting subsites when one more subsite to the aglycone side is added (as usual, we assume dimeric substrate and monomeric inhibitor). In this case, the binding of the inhibitor to subsite +3 competes with the nonproductive binding modes of the substrate (+2 to +3, and +1 to +2) but not with the productive binding (−1 to +1). Whether the competition of inhibitor with the nonproductive binding of substrate reveals as activation by inhibitor depends on the relative strengths of the productive- and nonproductive-binding modes of both, substrate and inhibitor. A more detailed analysis of enzymes with more than three subsites was beyond the scope of this study.

In a simplified approach, the inhibition is often measured at one substrate concentration and the results are presented in coordinates of *v*_*i*_/*v*_*i*=0_ versus [I], where *v*_*i*_ and *v*_*i*=0_ represent the initial rates measured in the presence and absence of inhibitor at concentration ([I]), respectively. Here, the inhibitory strength is expressed as IC_50_ value—the concentration of inhibitor at which the *v*_*i*_/*v*_*i*=0_ equals 0.5. For the three scenarios described above, the activating effect of inhibitor (*v*_*i*_/*v*_*i*=0_ values higher than 1) was seen only in the case of the first scenario (Fig. 2d). In the case of the second scenario [with *K*_si(np)_ = 5*K*_s_ and *K*_i(np)s_ = 5*K*_i(np)_], the activating effect revealed as a deviation from the “conventional” (consistent with hyperbola) decrease of *v*_*i*_/*v*_*i*=0_ values with the increasing inhibitor concentration. In the case of the third scenario, the *v*_*i*_/*v*_*i*=0_ versus [I] plots were consistent with the usual competitive inhibition. When present, the activating effect of inhibitor was more evident at high substrate concentrations (Fig. 2d). These results are expected, since the nonproductive binding has no influence on *k*_cat_/*K*_m_ (Fig. 1b), the parameter that governs the rate at low substrate concentrations.

### Transglycosylation to inhibitor

**Fig. 3:**
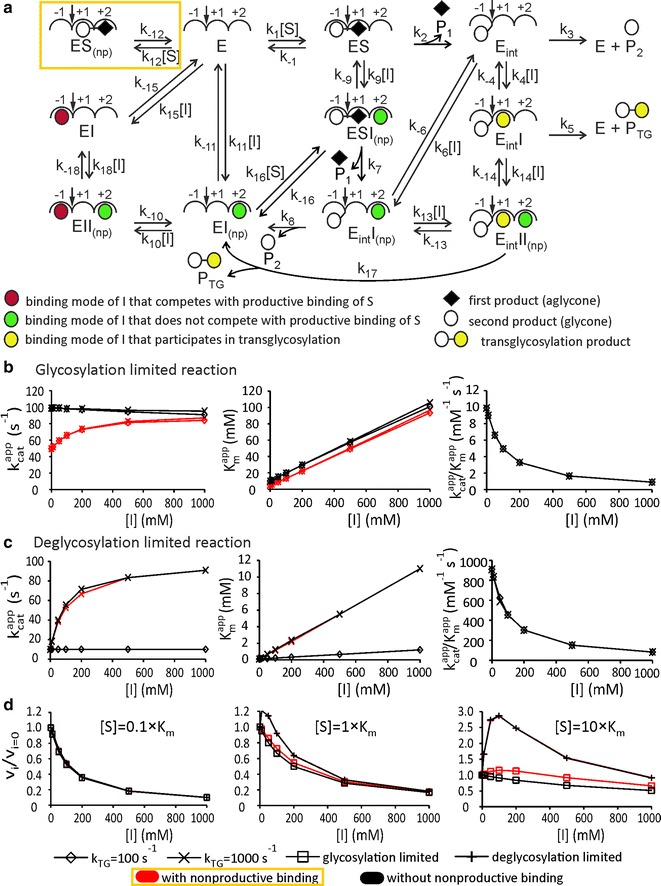
Effects of inhibitor to enzymes exerting the nonproductive binding of substrate and transglycosylation to inhibitor. **a** In this mechanism, a covalent glycosyl-enzyme intermediate (*E*_int_) is included. Rates of passing through elementary steps are represented by rate constants and concentration terms above corresponding* arrows*. After the formation (rate constants *k*_2_ and *k*_7_), *E*_int_ can break down by hydrolysis (rate constants *k*_3_ and *k*_8_) or by the transglycosylation to inhibitor (rate constants *k*_5_ and *k*_17_ collectively referred to as *k*_TG_). The steady-state solution was analyzed numerically. The values of the off-rate constants for the dissociation of the noncovalent complexes were set to 10^4^ and 10^5^ s^−1^ for the dissociation of substrate and inhibitor, respectively. The values of all the second-order rate constants were set to 10^3^ mM^−1^ s^−1^. **b** Glycosylation-limited reaction was mimicked by setting *k*_2_ = *k*_7_ = 100 s^−1^, and *k*_3_ = *k*_8_ = 10^4^ s^−1^. **c** Deglycosylation-limited reaction was mimicked by setting *k*_2_ = *k*_7_ = 10^5^ s^−1^, and *k*_3_ = *k*_8_ = 100 s^−1^. In both cases, the value of *k*_TG_ was set either 100 or 1000 s^−1^ as defined in the legend on figure. At both *k*_TG_ values, the analyses were made by assuming the presence or absence of the nonproductive binding of substrate (the complex in *yellow box* omitted). **d** Ratios of rates measured in the presence (*v*_*i*_) and absence (*v*_*i*=0_) of inhibitor as a function of inhibitor concentration. The concentration of substrate (as a multiple of its *K*_m_ value at [I] = 0) is shown in the plots. Note that there are four data series depicted in each plot on panels **b**–**d**, but some of them are not visible because of the overlap. All results presented are for the formation of the first product

Besides hydrolysis, many BGs have been shown to catalyze also transglycosylation reactions [8–10, 19, 60]. First, we consider the transglycosylation reactions involving inhibitors. With product inhibitors, a possible mechanism is the simple reverse reaction. The BGs most relevant in the context of cellobiose hydrolysis are those belonging to GH families 1 and 3. GHs within these families employ the retaining catalytic mechanism that involves a covalent glycosyl-enzyme intermediate. Therefore, the direct reverse reaction, i.e., between two product molecules, is unlikely in aqueous environment [60, 61]. The double displacement mechanism of retaining GHs can be divided into two steps. In the first step (glycosylation), the glycosidic bond oxygen is protonated to make the aglycone a better leaving group. In parallel, the catalytic nucleophile attacks the C1 of glycone resulting in the formation of the covalent glycosyl-enzyme intermediate. In the second step (deglycosylation), the intermediate is hydrolysed to release the glycone part of the substrate. Alternatively, the glycone can be released by the attack of the hydroxyl group of substrate (transglycosylation to substrate) or the sugar inhibitor (transglycosylation to inhibitor). The reaction scheme is depicted in Fig. 3a. To reduce the complexity of the rate equations, the transglycosylation to substrate was omitted. In addition, we assume that the binding subsites −1 and +1 are interacting and, therefore, the enzyme with nonproductively bound substrate is a dead-end complex (Fig. 3a). The mechanism in Fig. 3a was solved using steady-state treatment and software for the King Altman procedure [62]. The resulting complex equation was analyzed numerically. To single out the effects of the transglycosylation to inhibitor, we first analyzed the reaction mechanism without the nonproductive binding of substrate (the complex within yellow box is omitted). The values of the rate constants of enzyme glycosylation (*k*_2_ and *k*_7_) and deglycosylation by hydrolysis (*k*_3_ and *k*_8_) were set, so that they either did or did not support the plausibility of rapid equilibrium approach. To ensure that all complexes (except those involving the covalent intermediate) are at equilibrium, the glycosylation of enzyme should be much slower than both, deglycosylation of enzyme and all the dissociation steps of substrate and inhibitor. The situation was mimicked by setting *k*_2_ = *k*_7_ = 100 s^−1^, and *k*_3_ = *k*_8_ = 10^4^ s^−1^. The values of the off-rate constants for the dissociation of the noncovalent complexes were set to 10^4^ and 10^5^ s^−1^ for the dissociation of substrate and inhibitor, respectively. The values of all the second order rate constants were set to 10^3^ mM^−1^ s^−1^. The influence of transglycosylation was assessed by varying the rate constant of transglycosylation (*k*_TG_ = *k*_5_ = *k*_17_) between 100 and 10^4^ s^−1^. Without the presence of inhibitor, the numerical analyses resulted in the *k*_cat_ and *K*_m_ values of 99 s^−1^ and 10 mM, respectively. These figures are consistent with the input parameter values for *k*_2_ = *k*_7_ and the equilibrium dissociation constants of substrate (10^4^ s^−1^/10^3^ mM^−1^ s^−1^ = 10 mM). The *k*_cat_ of the first product formation was independent of the inhibitor concentration when the value of *k*_TG_ was set equal or higher than the rate constant for the hydrolysis of the glycosyl-enzyme intermediate (there was a slight decrease in *k*_cat_ with the increasing inhibitor concentration when *k*_TG_ was set lower than *k*_3_ = *k*_8_) (Fig. 3b). *K*_m_ increased linearly (there was a slight deviation from linearity at lower *k*_TG_ values) with the inhibitor concentration and was independent of the product used for rate measurements. The effects of inhibitor were characteristic to competitive inhibition. The *K*_i_ value found from the dependency of *K*_m_ from the inhibitor concentration was 100 mM and this matched the input values used for the dissociation equilibrium constants of inhibitor in the analysis (10^5^ s^−1^/10^3^ mM^−1^ s^−1^ = 100 mM). As expected, the *k*_cat_ for the second product formation decreased in parallel with the increasing of the *k*_cat_ for the formation of transglycosylation product with the increasing inhibitor concentration (Additional file 1: Figs. S2 and S3). Similar trends were observed when *k*_TG_ was kept constant (10^3^ s^−1^) and the binding strength of inhibitor to the glycosyl-enzyme intermediate was varied between 1.0 and 100 mM (data not shown). Taking together, the activation by transglycosylation to inhibitor is not observed when the enzyme glycosylation is rate limiting for the glycosidic bond hydrolysis. This is expected, since transglycosylation can boost only the rate of deglycosylation and has, hence, no effect on the rate-limiting step.

Next, we analyzed the effects of the transglycosylation to inhibitor in the conditions of enzyme deglycosylation being the rate-limiting step (again, we first ignore the nonproductive binding by omitting the complex within yellow box in Fig. 3a). The situation was mimicked by setting *k*_2_ = *k*_7_ = 10^5^ s^−1^, and *k*_3_ = *k*_8_ = 100 s^−1^. The values of the rest of the rate constants were kept as in the case of the analysis of the glycosylation-limited reaction (see above). Without inhibitor, the values of 99 s^−1^ and 0.11 mM were found for *k*_cat_ and *K*_m_, respectively. About two orders of magnitude, lower *K*_m_ (compared to the true equilibrium dissociation constants) reflects the contribution of the glycosyl-enzyme intermediate complexes in the substrate binding. The increase of the *k*_cat_ of the first product formation with the increasing inhibitor concentration was observed when the value of *k*_TG_ was set higher than 100 s^−1^. *K*_m_ also increased with the increasing inhibitor concentration. However, *k*_cat_/*K*_m_ decreased with the increasing inhibitor concentration and was independent of the value of *k*_TG_ (Fig. 3b). The same trends were observed in the series with constant *k*_TG_ (1000 s^−1^) and varied binding strength of inhibitor to the glycosyl-enzyme intermediate complexes (between 1.0 and 100 mM) (data not shown). Thus, the transglycosylation to inhibitor was able to explain the increase in *k*_cat_ with the increasing inhibitor concentration only when the rate limiting step for the glycosidic bond hydrolysis was the enzyme deglycosylation. In both cases, the glycosylation and deglycosylation-limited reaction, transglycosylation had no effect on *k*_cat_/*K*_m_ of the first product formation.

In the second part of this subtopic, we analyzed the combined effects of the transglycosylation and nonproductive binding of substrate (Fig. 3a, complex within the yellow box included). The dissociation rate constant of the enzyme-substrate nonproductive complex was set equal to that used for the productive complexes, i.e., 10 mM (10^4^ s^−1^/10^3^ mM^−1^ s^−1^). All the other conditions were exactly the same as described in the first part of this subtopic (see above). First, we analyzed the glycosylation-limited reaction (*k*_2_ = *k*_7_ = 100 s^−1^, and *k*_3_ = *k*_8_ = 10^4^ s^−1^). Without the presence of inhibitor, the numerical analyses resulted in the *k*_cat_ and *K*_m_ values of 49.5 s^−1^ and 5 mM, respectively. Both values were reduced by the factor of 2 when compared to the equivalent case without the nonproductive binding of substrate. This result is expected, since the strengths of both productive- and nonproductive binding modes were set equal (see equations in Figs. 1 and 2b). The *k*_cat_ of the first product formation increased with the increasing inhibitor concentration and approached the value of the rate constant of the enzyme glycosylation when *k*_TG_ was set high (Fig. 3b). This is different from the case without the nonproductive binding, where there was no effect of inhibitor on *k*_cat_ in the glycosylation-limited reaction. Like in case without the nonproductive binding, here also, *K*_m_ increased and *k*_cat_/*K*_m_ decreased with the increasing inhibitor concentration (Fig. 3b). Next, we set the values of the rate constants to mimic the deglycosylation-limited reaction (*k*_2_ = *k*_7_ = 10^5^ s^−1^, and *k*_3_ = *k*_8_ = 100 s^−1^). Without the presence of inhibitor, the values of 99 s^−1^ and 0.11 mM were calculated for *k*_cat_ and *K*_m_, respectively. These figures match the corresponding values found in the analyses without the nonproductive binding, indicating that in the case of the deglycosylation-limited reaction, the nonproductive binding has no effect on *k*_cat_ and *K*_m_. This result is expected, since the accumulation of glycosyl-enzyme intermediate can increase only the apparent strength of the productive binding mode (see equations in Fig. 1 with *K*_m_^exp^/*K*_s(np)_ close to zero). Because of the negligible contribution of the nonproductive binding in the case of the deglycosylation-limited reaction, all effects of inhibitor were the same as described in the first part of this subtopic (the deglycosylation-limited reaction without the nonproductive complex).

Finally, we analyzed the data in the coordinates of *v*_*i*_/*v*_*i*=0_ versus [I]. In all cases, the activating effect of inhibitor was evident only at substrate concentrations higher than *K*_m_ (Fig. 3d). This is expected, since neither the nonproductive binding nor the transglycosylation to inhibitor had any effect on *k*_cat_/*K*_m_. The activating effect caused by the transglycosylation to inhibitor appears stronger than that caused by the competition with nonproductive binding (Fig. 3d). This is because the value of *k*_TG_ was set tenfold higher than the value of the rate constant for deglycosylation by hydrolysis, but the strength of the nonproductive binding was set equal to the strength of the productive binding (for the opposite case, see Additional file 1: Fig. S1).

### Transglycosylation to substrate

**Fig. 4:**
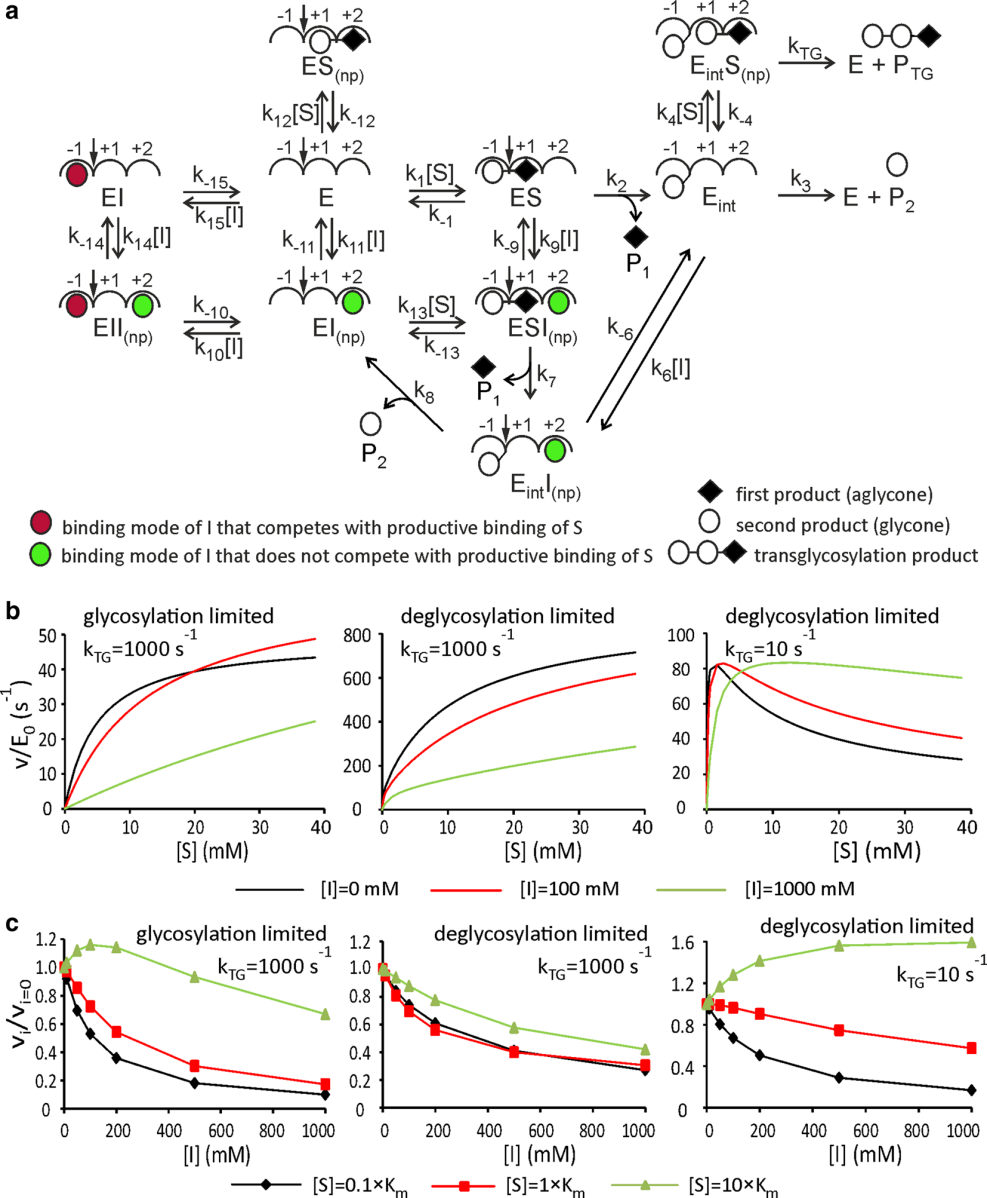
Effects of inhibitor to enzymes exerting the nonproductive binding of substrate and transglycosylation to substrate. **a** In this mechanism, a covalent glycosyl-enzyme intermediate (*E*_int_) is included. Rates of passing through the elementary steps are represented by rate constants and concentration terms above corresponding* arrows*. After the formation (rate constants *k*_2_ and *k*_7_), *E*_int_ can break down by hydrolysis (rate constants *k*_3_ and *k*_8_) or by the transglycosylation to substrate (rate constant *k*_TG_). The steady-state solution was analyzed numerically. The values of the off-rate constants for the dissociation of the noncovalent complexes were set to 10^4^ and 10^5^ s^−1^ for the dissociation of substrate and inhibitor, respectively. The values of all the second-order rate constants were set to 10^3^ mM^−1^ s^−1^. **b** The dependency of the ratio of steady-state velocity to the total enzyme concentration from the concentration of substrate. **c** Ratios of rates measured in the presence (*v*_*i*_) and absence (*v*_*i*=0_) of inhibitor as a function of the inhibitor concentration. The concentration of substrate (as a multiple of its *K*_m_ value at [I] = 0, or as a multiple of its concentration at optimum in the case of the rightmost plot) is shown in the legend on figure. In panels **b** and **c,** the glycosylation-limited reaction was mimicked by setting *k*_2_ = *k*_7_ = 100 s^−1^, and *k*_3_ = *k*_8_ = 10^4^ s^−1^. The deglycosylation-limited reaction was mimicked by setting *k*_2_ = *k*_7_ = 10^5^ s^−1^, and *k*_3_ = *k*_8_ = 100 s^−1^. In both cases, the value of *k*_TG_ was set either 10 s^−1^ or 1000 s^−1^ as defined in the plots. All results presented are for the formation of the first product

The transglycosylation to substrate is often observed with BGs. The transglycosylation to substrate has been demonstrated to be responsible for the substrate inhibition of BGs, a phenomenon where Michaelis–Menten equation breaks down [9, 10]. At low substrate concentrations, the velocity increases with increasing substrate concentration but starts to decrease when substrate concentration exceeds a certain optimum concentration. The activation of BGs that show substrate inhibition (i.e., non Michaelis–Menten kinetics) by inhibitor has also reported [32, 38]. Therefore, we were curious whether the competition of inhibitor with the nonproductive binding of substrate can result in the activation of enzyme also in the presence of substrate inhibition. A possible mechanism is depicted in Fig. 4a. The mechanism is reminiscent to that depicted in Fig. 3a, but the transglycosylation to inhibitor was omitted. Therefore, the scheme in Fig. 4a assumes that the binding of the inhibitor to subsite +1 is weak compared to the binding to subsites −1 and +2, so that its contribution is insignificant. Like in Fig. 3a, we also assumed here fully interacting subsites −1 and +1 (except when there is a covalent glycosyl-enzyme intermediate in −1) and noninteracting subsites +1 and +2. Three scenarios were addressed using numerical analysis: (1) glycosylation-limited reaction, (2) deglycosylation-limited reaction with the rate constant for the breakdown of glycosyl-enzyme intermediate by the transglycosylation to substrate (*k*_TG_) higher than that by hydrolysis, and (3) deglycosylation-limited reaction with *k*_TG_ lower than the rate constant for the hydrolysis of glycosyl-enzyme (*k*_3_ and *k*_8_). The values of rate constants were set to mimic glycosylation or deglycosylation-limited reaction exactly as described for the analysis of scheme in Fig. 3a. The values of all second-order rate constants were set to 10^3^ mM^−1^ s^−1^. The values of off-rate constants for the dissociation of noncovalent complexes were set to 10^4^ and 10^5^ s^−1^ for the dissociation of substrate and inhibitor, respectively. Consequently, all equilibrium dissociation constants for substrate had a value of 10 mM and those for inhibitor 100 mM. If not stated otherwise, we discuss below the kinetics of the formation of the first product. In the case of the glycosylation-limited reaction (the first scenario), the kinetics was consistent with Michaelis–Menten equation (except in case *k*_TG_ = 0, where a slight substrate inhibition was present). The *k*_cat_ and *K*_m_ values were independent of the value of *k*_TG_ (data not shown). This is expected, since the transglycosylation to substrate cannot boost the rate-limiting glycosylation step. Since the glycosyl-enzyme intermediate does not accumulate in the glycosylation-limited reaction, no significant substrate inhibition is expected (except the binding of substrate to the glycosyl-enzyme intermediate is much stronger than the other binding modes of substrate). The kinetics was consistent with Michaelis–Menten equation also in the presence of inhibitor (Fig. 4b). *k*_cat_ increased with increasing inhibitor concentration, but *k*_cat_/*K*_m_ always decreased. In short, the kinetics was similar to that captured by mechanism in Fig. 3a in the case of glycosylation-limited reaction and the presence of nonproductive binding. In the case of deglycosylation-limited reaction (the second scenario), the Michaelis–Menten equation holds as far as the value of *k*_TG_ is set equal or higher than the value of the rate constant for the hydrolysis of glycosyl-enzyme intermediate (Fig. 4b). At *k*_TG_ values much higher than hydrolysis, the enzyme is effectively a glycosynthase and the second product is not formed. In the presence of inhibitor, the kinetics deviated from the Michaelis–Menten equation (Fig. 4b), but there were no activating effects of inhibitor. This is consistent with the results from the analysis of mechanism in Fig. 3a. The activating effect resulting from the competition of inhibitor with the nonproductive binding had no effect in the case of the deglycosylation-limited reaction (Fig. 3) and the competition of inhibitor with the binding of substrate to the glycosyl-enzyme intermediate can only inhibit when *k*_TG_ is high. In the case of the deglycosylation-limited reaction but the value of *k*_TG_ lower than that of the corresponding constant for hydrolysis (the third scenario), there was a characteristic substrate inhibition in coordinates of v versus [S] (Fig. 4b). The optimal substrate concentration shifted towards higher concentration values with increasing inhibitor concentration. A clear activating effect of inhibitor was evident at higher substrate concentrations (Fig. 4b).

In the coordinates of *v*_*i*_/*v*_*i*=0_ versus [I], the activating effect of inhibitor was seen for the first and third scenario (Fig. 4c). Similarly to all cases discussed above in this study, the activating effect of inhibitor was best evident at substrate concentrations above *K*_m_ (or above optimal substrate concentration in the case of the third scenario). In the case of the glycosylation-limited reaction, the activating effect was caused by the competition of inhibitor with the nonproductive binding of substrate. In the case of the deglycosylation-limited reaction, the activating effect was caused by the competition of inhibitor with the binding of substrate to the glycosyl-enzyme intermediate (provided that the value of *k*_TG_ is lower than that of hydrolysis).

## Discussion

BGs often show a complex kinetics, including inhibitory effects of substrate and activating effects of inhibitors. The substrate inhibition caused by the competing hydrolysis and transglycosylation to substrate reactions is well recognized [8–10]. This type of substrate inhibition is easily detected because of the breakdown of Michaelis–Menten saturation kinetics. Another inhibitory effect of substrate can be seen in nonproductive binding, which competes with the productive binding of substrate. Since, in this case, the Michaelis–Menten saturation kinetics holds, the effects of nonproductive binding are often overlooked. A kinetic peculiarity of many BGs is the activation of enzyme by inhibitor at low-to-moderate concentrations followed by inhibition at high concentrations. The most common explanation to this phenomenon is the transglycosylation to inhibitor, and, indeed, in many cases, the transglycosylation products are observed in reactions containing inhibitor [19, 26]. However, the activation by inhibitor has been reported also for BGs which do not produce transglycosylation products with inhibitors [38]. The competition of glucose with substrate for binding to the enzyme-substrate complex has recently been proposed to be responsible for the glucose activation of a mutant BG of *T. reesei* [38]. The activation by inhibitor has also been explained by assuming the presence of an allosteric regulatory binding site for inhibitor [21, 39]. In this study, we demonstrate that activation by inhibitor can be accounted for by a simple competition of inhibitor with the nonproductive binding of substrate without assuming any allosteric effects or conformational changes in protein (Figs. 2, 3, 4). Obviously, the transglycosylation to inhibitor was also able to account for the activation when the rate constant for transglycosylation reaction was higher than that for hydrolysis (Fig. 3). In both cases, the activation was manifested by the increase in apparent *k*_cat_ with the increasing inhibitor concentration. The increase in apparent *K*_m_ counterbalanced the increase in *k*_cat,_ so that *k*_cat_/*K*_m_ always decreased with increasing inhibitor concentration. Therefore, the activating effect was seen only at substrate concentrations around or above *K*_m_ when studied in coordinates of *v*_*i*_/*v*_*i*=0_ versus [I] (Figs. 2, 3, 4). Such an increase of the activating effect of glucose and xylose with the increasing pNPG concentration has been described for GH1 BG of *Humicola insolens* [21]. In the literature, the increase in *k*_cat_ and *K*_m_ of BGs with increasing glucose concentration has been reported [19, 21, 26, 30, 63]. In some studies, the decrease of *k*_cat_/*K*_m_ with increasing glucose concentrations is observed [26], whereas in others, a little change or a slight increase of *k*_cat_/*K*_m_ is observed [19, 21]. The effects of inhibitors on *k*_cat_/*K*_m_ also seem to depend on the nature of sugar inhibitors [19] and substrate [63]. Although detailed studies of allosteric effects were beyond the scope of the present work, we note that the transglycosylation to inhibitor can result in the increase of *k*_cat_/*K*_m_ with increasing inhibitor concentration when the presence of separate “transglycosylation-mode”-binding site is assumed for inhibitor. In these analyses, we further assumed that the transglycosylation-mode-binding site is accessible to inhibitor only in the productive enzyme-substrate complex and not in the other enzyme forms (Additional file 1: Fig. S4).

The BGs showing glucose activation belong to a group of BGs referred to as glucose tolerant BGs. These BGs are mostly, but not exclusively, gathered to the GH family 1. Compared to the shallow substrate-binding cleft of GH3 BGs, the active site of GH1 BGs rests in the bottom of a deep and narrow cavity [40]. Such active site architecture has been proposed to limit the access of glucose to the glycone-binding site and confer the glucose tolerance [40]. Whatever is the underlying mechanism, the amino-acid residues responsible for the glucose tolerance are proposed to be located in the aglycone-binding site [40, 64, 65]. In the context of the effects of the nonproductive binding of substrate reported here, it is important that the structures of GH1 BGs show the presence of more than one aglycone-binding subsites [30, 40, 66–68]. In the structure of *H. insolens* BG in complex with glucose, the glucose is bound at the aglycone-binding site +2, suggesting the strongest interaction with glucose in this subsite [40]. The strongest interaction with glucose unit in +2 subsite is also reported with rice BGs [67, 69]. Strong interactions with glucose in subsite +2 are expected to result in the nonproductive binding of substrate and thus a possible activation by glucose through the competition with nonproductive binding. With this mechanism, it is expected that mutations that reduce the binding strength in +2 should reduce the nonproductive binding of substrate and hence increase both *k*_cat_ and *K*_m_ with no change in *k*_cat_/*K*_m_. We note as a caveat here that the contribution of the competition of inhibitor with the nonproductive binding of substrate in the inhibitor activation not only depends on the relative affinities of the different binding modes but also on whether and which subsites are interacting (Fig. 2). Regardless of other plausible alternatives, it is tempting to speculate that alterations in the nonproductive binding of substrate upon mutations are at least partly responsible for the effects of mutations far from the catalytic residues observed with many BGs [55, 63, 70–73]. Contrarily to *H. insolens* BG in the structures of BG Td2F2 [30], and BG of *Paenibacillus polymyxa* [66] glucose is found in the binding site −1. With Td2F2, the transglycosylation to inhibitor has been proposed to be responsible for glucose activation [19, 30]. The unequivocal evidence for the contribution of transglycosylation can be provided by measuring of the concentrations of all hydrolysis and transglycosylation products. This has been done for some fungal BGs, but only the transglycosylation to substrate and not to inhibitor was addressed [9, 10]. To the best of our knowledge, the rigorous quantitative analysis of the products resulting from the transglycosylation to inhibitor (glucose) has been made only for BG Td2F2 [19]. The analyses made here suggest that the mechanism of the inhibitor activation is related to the rate-limiting step in the absence of inhibitor. The transglycosylation to inhibitor did not result in activation when the rate-limiting step was enzyme glycosylation. In this case, the activation could be explained by the competition of inhibitor with the nonproductive binding of substrate. The opposite was true when the rate-limiting step was enzyme deglycosylation (hydrolysis of the covalent glycosyl-enzyme intermediate). Using experiment set-up where only the hydrolysis is possible, there is no concentration dependencies, and the nature of the rate-limiting step cannot be assessed using kinetics measurements at steady-state. However, the nature of the rate-limiting step can be revealed by including measurements in the pre-steady-state regime. In these studies, the chromogenic model substrates, like pNP-sugar derivatives, are often used. In the case of fast glycosylation and slow deglycosylation (more precisely, a slow step after glycosylation), the initial release of aglycone follows the so-called burst [42]. BG from *Agrobacterium circulans* shows no burst in the release of aglycone when studied with pNPG substrate, suggesting that the glycosylation is the rate limiting step [74]. Similar conclusions have been made for the xylanase of *Bacillus circulans* [75] and α-amylase from *Bacillus subtilis* [76]. It is also worth noting that the glycosylation-limited reaction of a wild-type enzyme can be turned to a deglycosylation limited by the mutagenesis of enzymes [74, 75]. Using the steady-state measurements and concentration dependencies (transglycosylation to substrate), Bohlin et al. found the rate constants for the formation of glycosyl-enzyme intermediate and its breakdown by hydrolysis and transglycosylation for six fungal, GH family 3, BGs [9]. With all BGs, the values of glycosylation rate constants were lower than those of deglycosylation by hydrolysis and transglycosylation. However, the difference between the rate constants was not large enough to assign the glycosylation as the sole rate determining step [9]. When both, glycosylation and deglycosylation, steps have significant contribution in controlling the rate, the activating effects by inhibitor are also expected to involve both, the effects of competition of inhibitor with the nonproductive binding of substrate and the effects of the transglycosylation to inhibitor. Clearly, more detailed kinetic and structural studies are required for uncovering the mechanisms of the activation of different BGs by inhibitors.

## Conclusions

The activation by inhibitor at lower and inhibition at higher inhibitor concentration is often seen in studies of the glucose inhibition of BGs. Here, we demonstrate that this phenomenon can be accounted for by a simple competition of inhibitor with the nonproductive binding of substrate. In addition, the transglycosylation to inhibitor was able to account for the activation by inhibitor. With both mechanisms, the activation was caused by the increase of *k*_cat_ with the increasing inhibitor concentration. However, *k*_cat_/*K*_m_ always decreased with the increasing inhibitor concentration, unless the presence of an allosteric regulatory binding site for inhibitor was assumed. The possible contribution of different mechanisms in the activation by inhibitor was found to be dependent on the rate-limiting step. The transglycosylation to inhibitor did not result in activation when the rate-limiting step was enzyme glycosylation. In this case, the activation could be explained by the competition of inhibitor with the nonproductive binding of the substrate. The opposite was true for the enzyme deglycosylation-limited reaction. The contribution of different mechanisms was further found to be dependent on whether and which glucose-unit-binding subsites are interacting. Distinguishing between activation mechanisms is important in the point of view of the application of BGs in aiding cellulose saccharification. When activation is caused by transglycosylation to inhibitor, it is important to consider the possible inhibition of enzymes in cellulolytic cocktail by transglycosylation products. This is not an issue when the competition between inhibitor and the nonproductive binding of substrate is responsible for the activation of BG.

## Methods

The mechanism in Fig. 2 was solved using rapid equilibrium treatment. The mechanisms in Figs. 1, 3, and 4 were solved using the steady-state treatment. For the mechanisms in Figs. 3 and 4, the software for the King Altman procedure [62] was used to derive the rate equations. Numerical analyses were performed using software Microsoft Excel.

## Additional file

**Additional file 1: Figure S1.** Effect of inhibitor to an enzyme exerting the nonproductive binding of substrate. **Figure S2**. Effects of inhibitor to enzymes exerting the nonproductive binding of substrate and transglycosylation to inhibitor-formation of the second product. **Figure S3**. Effects of inhibitor to enzymes exerting the nonproductive binding of substrate and transglycosylation to inhibitor-formation of the transglycosylation product. **Figure S4**. Effects of inhibitor to enzymes exerting the transglycosylation to inhibitor that binds to the “transglycosylation-binding-site”.

## Abbreviations

BG: β-glucosidase
GH: glycoside hydrolase
pNPG: para-nitrophenol-β-glucoside
