# Supplementary material for: When substrate inhibits and inhibitor activates: implications of β-glucosidases
Source: Biotechnol Biofuels. 2017 Jan 3;10:7. doi: 10.1186/s13068-016-0690-z (PMC5209912; doi:10.1186/s13068-016-0690-z)
Supplement: Supplementary file 1 — Additional file 1: Figure S1. Effect of inhibitor to an enzyme exerting the nonproductive binding of substrate. Figure S2. Effects of inhibitor to enzymes exerting the nonproductive binding of substrate and transglycosylation to inhibitor-formation of the second product. Figure S3. Effects of inhibitor to enzymes exerting the nonproductive binding of substrate and transglycosylation to inhibitor-formation of the transglycosylation product. Figure S4. Effects of inhibitor to enzymes exerting the transglycosylation to inhibitor that binds to the “transglycosylation-binding-site”. [file 13068_2016_690_MOESM1_ESM.docx]

**Supplemental material to:**

**When substrate inhibits and inhibitor activates – implications of β-glucosidases**

Silja Kuusk and Priit Väljamäe*


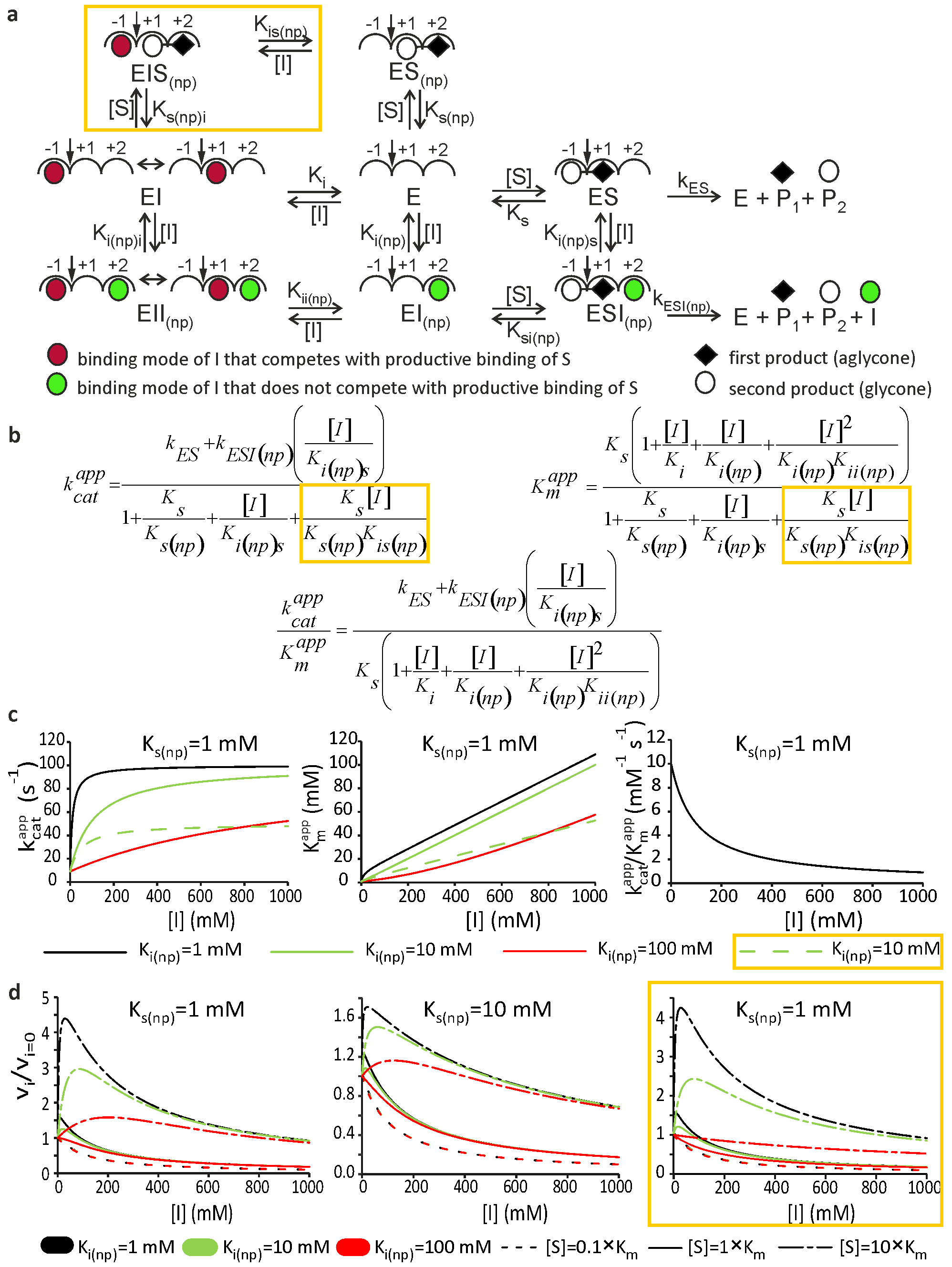


**Fig. S1** Effect of inhibitor to an enzyme exerting the nonproductive binding of substrate. **a** The binding of inhibitor (I) to the binding site +2 (designated as the nonproductive (np) binding mode of inhibitor) competes with nonproductive binding but not with the productive binding of substrate. The binding of inhibitor to subsites –1 or +1 competes with the productive binding of substrate. For simplicity these binding modes are lumped together (designated by double-headed arrow) in EI and EII_(np)_ complexes. The binding of inhibitor to +1 competes also with the nonproductive binding of substrate, whereas the binding to –1 does not. The latter possibility is accounted for by the presence of EIS_(np)_ complex (within yellow box). The stability of all complexes is represented by the corresponding equilibrium dissociation constants. **b** Michaelis Menten equation (Eq. 2) parameters derived using the equilibrium treatment. The same equations are applicable for the formation of the first, and the second product as well as for the disappearance of substrate. The terms within yellow boxes appear only when the EIS_(np)_ complex is included into the reaction scheme. The scheme and the equations are exactly the same as those in Fig.2 of the main article. **c** The dependency of parameters on the inhibitor concentration. **d** The ratios of the rates measured in the presence (v_i_) and absence (v_i=0_) of inhibitor as a function of the inhibitor concentration. The concentration of substrate (as a multiple of its *K*_m_ value at [I]=0) is shown in the plots. In generating the data for panels c and d the following values of the rate constants and equilibrium dissociation constants were used in analyses: *k*_ES_=*k*_ESI(np)_=100 s^-1^, *K*_s_=*K*_si(np)_=10 mM, *K*_i_=*K*_ii(np)_=*K*_i(np)i_=*K*_is(np)_=100 mM. The *K*_s(np)_ value was set equal to the *K*_s(np)i_ value and is shown in the plots. The *K*_i(np)_ value was set equal to the *K*_i(np)s_ value and is shown with different line colors. Data labels and plots within the yellow box correspond to the mechanism including the EIS_(np)_ complex.

**
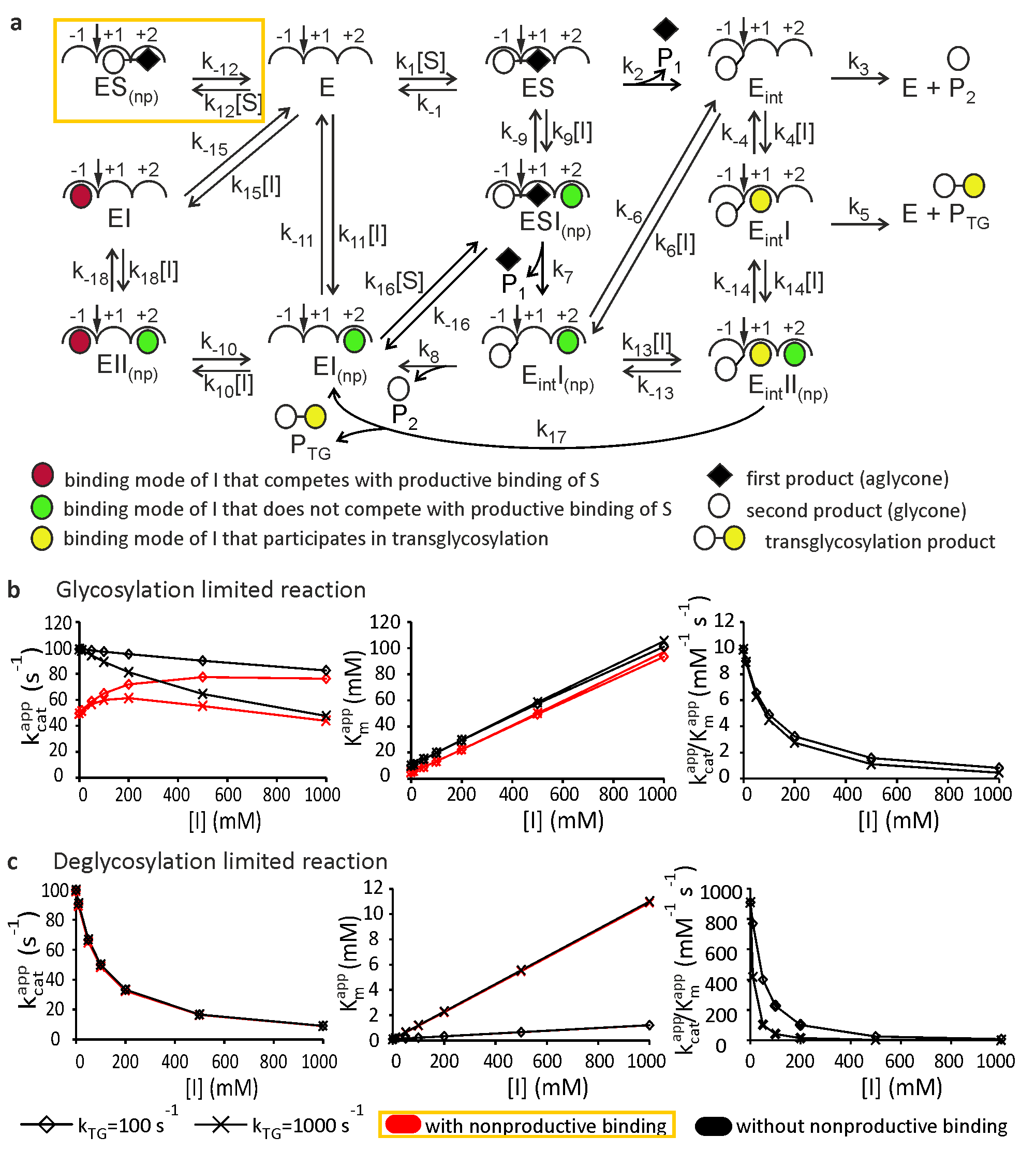
Fig. S2**. Effects of inhibitor to enzymes exerting the nonproductive binding of substrate and transglycosylation to inhibitor. **a** In this mechanism a covalent glycosyl-enzyme intermediate (E_int_) is included. The rates of passing through elementary steps are represented by the rate constants and concentration terms above the corresponding arrows. After formation (rate constants *k*_2_ and *k*_7_), E_int_ can break down by hydrolysis (rate constants *k*_3_ and *k*_8_) or by the transglycosylation to inhibitor (rate constants *k*_5_ and *k*_17_ collectively referred to as *k*_TG_). The steady state solution was analyzed numerically. The values of the off-rate constants for the dissociation of the non-covalent complexes were set to 10^4^ s^-1^ and 10^5^ s^-1^ for the dissociation of substrate and inhibitor, respectively. The values of all the second order rate constants were set to 10^3^ mM^-1^ s^-1^. **b** The glycosylation limited reaction was mimicked by setting *k*_2_=*k*_7_=100 s^-1^, and *k*_3_=*k*_8_=10^4^ s^-1^. **c** The deglycosylation limited reaction was mimicked by setting *k*_2_=*k*_7_=10^5^ s^-1^, and *k*_3_=*k*_8_=100 s^-1^. In both cases, the value of *k*_TG_ was set either 100 s^-1^ or 1000 s^-1^ as defined on the figure. At both *k*_TG_ values the analyses were made by assuming the presence or absence of the non-productive binding of substrate (complex in yellow box omitted). Note that there are four data series depicted in each plot on panels b and c, but some of them are not visible because of the overlap. All results presented are for the formation of the second product.


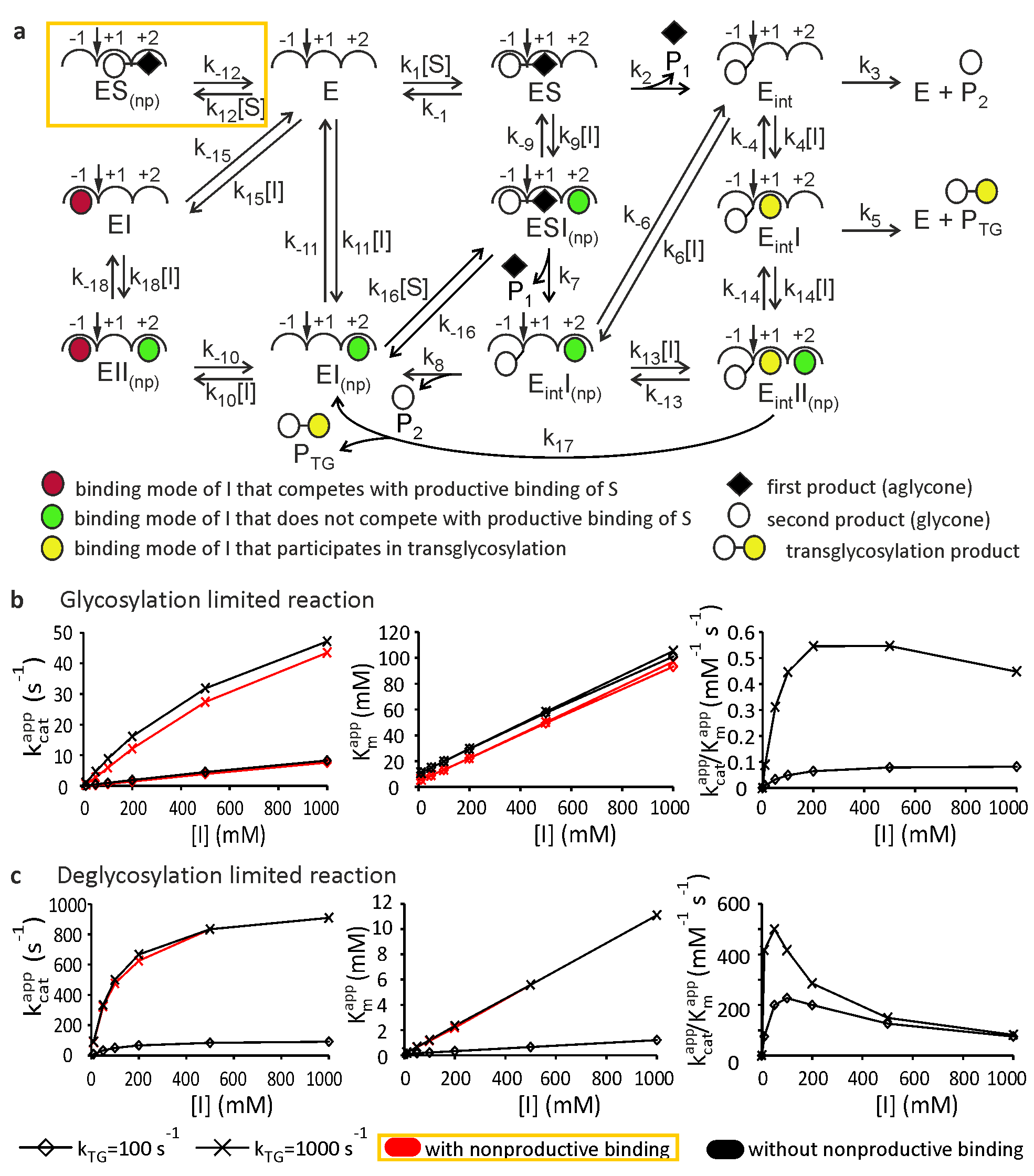


**Fig. S3**. Effects of inhibitor to enzymes exerting the nonproductive binding of substrate and transglycosylation to inhibitor. **a** In this mechanism a covalent glycosyl-enzyme intermediate (E_int_) is included. The rates of passing through the elementary steps are represented by the rate constants and concentration terms above the corresponding arrows. After the formation (rate constants *k*_2_ and *k*_7_), E_int_ can break down by hydrolysis (rate constants *k*_3_ and *k*_8_) or by the transglycosylation to inhibitor (rate constants *k*_5_ and *k*_17_ collectively referred to as *k*_TG_). The steady state solution was analyzed numerically. The values of off-rate constants for the dissociation of the non-covalent complexes were set to 10^4^ s^-1^ and 10^5^ s^-1^ for the dissociation of substrate and inhibitor, respectively. The values of all the second order rate constants were set to 10^3^ mM^-1^ s^-1^. **b** The glycosylation limited reaction was mimicked by setting *k*_2_=*k*_7_=100 s^-1^, and *k*_3_=*k*_8_=10^4^ s^-1^. **c** The deglycosylation limited reaction was mimicked by setting *k*_2_=*k*_7_=10^5^ s^-1^, and *k*_3_=*k*_8_=100 s^-1^. In both cases, the value of *k*_TG_ was set either 100 s^-1^ or 1000 s^-1^ as defined on the figure. At both *k*_TG_ values the analyses were made by assuming the presence or absence of the non-productive binding of substrate (complex in yellow box omitted). Note that there are four data series depicted in each plot on panels b and c, but some of them are not visible because of the overlap. All results presented are for the formation of the transglycosylation product.


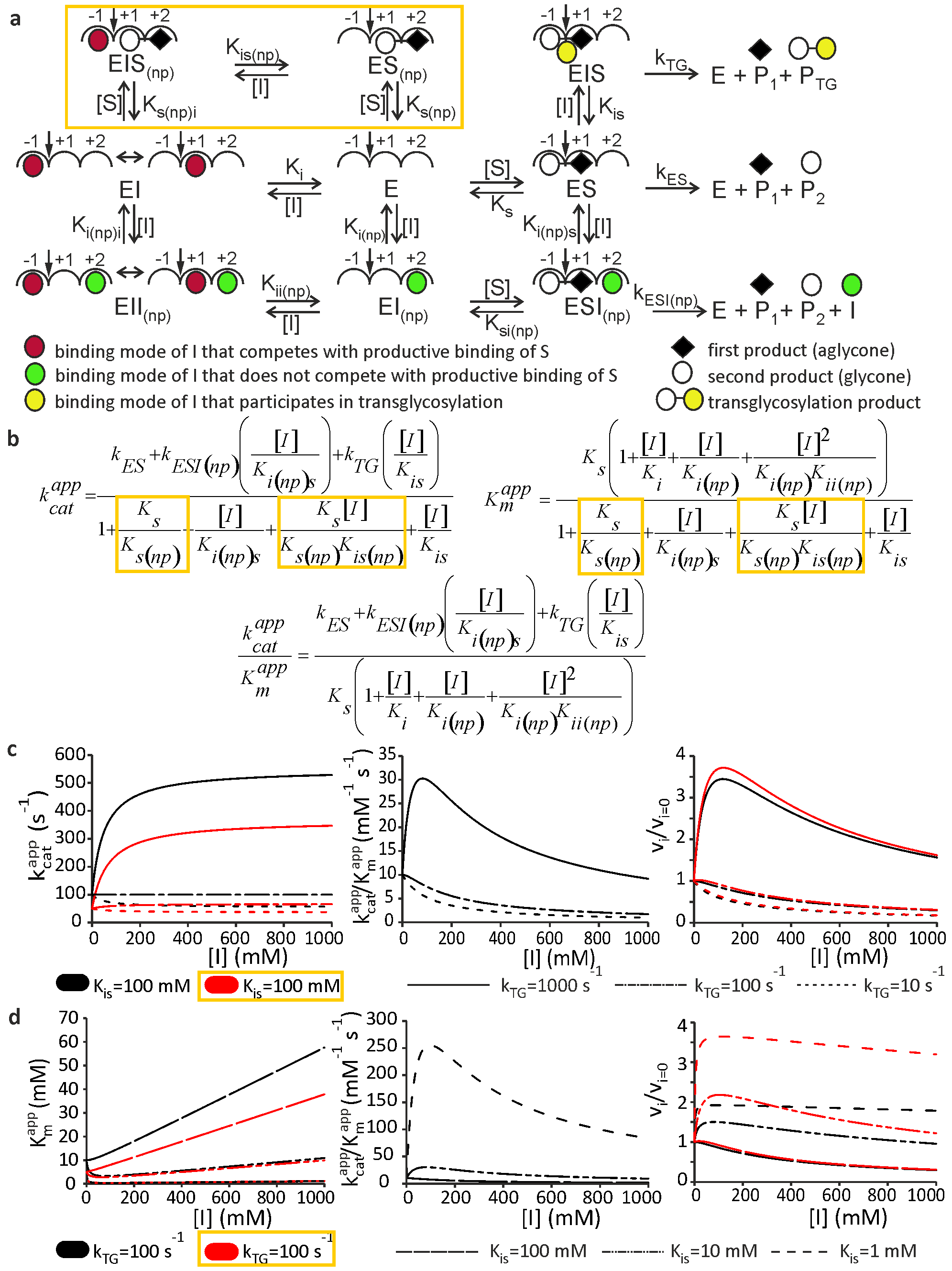


**Fig. S4** Effects of inhibitor to enzymes exerting the transglycosylation to inhibitor and the nonproductive binding of substrate. **a** The binding of inhibitor to the allosteric “transglycosylation binding site” on the enzyme substrate complex results in the formation of EIS complex which breaks down in the transglycosylation reaction with the rate constant *k*_TG_. The complexes within yellow box appear only in the case of the nonproductive binding of substrate. The rest is same as in the mechanism in Fig. 2b. **b** The Michaelis Menten equation parameters derived using the equilibrium treatment. The equations correspond to the formation of the first product. The terms within yellow boxes appear only in the case when the nonproductive binding of substrate is included. **c** Effects of the value of the rate constant for transglycosylation (*k*_TG_, shown with different line style) with *K*_is_ set to 100 mM. **d** Effects of the value of equilibrium dissociation constant of the transglycosylation complex (*K*_is_) with *k*_TG_ set 100 s^-1^. In generating the data for panels c and d the following values of rate constants and equilibrium dissociation constants were used in analyses: *k*_ES_=*k*_ESI(np)_=100 s^-1^, *K*_s_=*K*_si(np)_=*K*_s(np)_=*K*_s(np)i_=10 mM, *K*_i_=*K*_ii(np)_=*K*_i(np)i_=*K*_is(np)_=*K*_i(np)_=*K*_i(np)s_=100 mM. In v_i_/v_i=0_ *versus* [I] plots the concentration of substrate was set equal to its *K*_m_ value at [I]=0. Data labels within the yellow box correspond to the mechanism including the nonproductive binding of substrate.
